# Reliability analysis of the compressed air supplying system in underground mines

**DOI:** 10.1038/s41598-023-33736-5

**Published:** 2023-04-26

**Authors:** Mohammad Javad Rahimdel, Behzad Ghodrati

**Affiliations:** 1 Department of Mining Engineering, Faculty of Engineering, University of Birjand, Birjand, Iran; 2 Division of Operation and Maintenance Engineering, Lulea University of Technology, Lulea, Sweden

**Keywords:** Mechanical engineering, Civil engineering

## Abstract

Despite the high cost and low efficiency, compressed air is mostly used in underground mining for ore extraction, hoisting, and mineral processing operations. Failures of compressed air systems not only threaten the health and safety of workers but also contribute to inefficient control of airflow and stopped all equipment that operates by compressed air. In such uncertain conditions, mine managers are faced with the big challenge to supply enough compressed air, and therefore, the reliability evaluation of these systems is essential. This paper aims to analyze the reliability of the compressed air system using the Markov modeling approach as a case study, Qaleh-Zari Copper Mine, Iran. To achieve this, the state space diagram was constructed considering all relevant states for all compressors in the main compressor house of the mine. The failure and repair rate of all main and reserve compressors were calculated for all possible transitions between states to obtain the probability of being of the system in each of the states. Moreover, the probability of failure at any time period was considered to study the reliability behavior. The results of this study show that there is 31.5% probability that the compressed air supplying system is in operating condition with two main and one standby compressors. The system probability that two main compressors are remain in the operation without failure for one months is 92.32%. Furthermore, the lifetime of the system is estimated 33 months when at least one main compressor is active.

## Introduction

Both hydraulic and pneumatic powers are widely used to supply energy for mining equipment. Despite the high cost and low efficiency, compressed air is still mostly used in different equipment such as drilling pickers, pumps, fans, turbine lights, cranes, conveyors, loaders, and excavators. Furthermore, it is usually used for the sake of ventilation door operations and water spraying to dust diminishing. The compressors are needed to be active continuously to keep the adequate system pressure. The compressed air supply system is one of the most energy-consuming systems in mine contributes about 20–40% of the total energy consumption of mine^1^. Like any other system, air compressor units are formed from different parts including tanks (air deposit), hoses, pipes, cables, etc. These parts are regularly exposed to distraction and erosions. There is a possibility of revealing the damage before the occurrence of any type of erosions. However, any failure of the compressed air supplying system exposes the operators and equipment to a high level of safety risk.

The use of positive displacement compressors (screw and piston compressors), which are capable to supply high-pressure compressed air is mostly routine in underground mining operations. Trusting just one compressor is highly risky since first of all being dependent on only one compressor may bring in the capability of supplying the airflow of pressurized air which is needed and secondly, the failure of the device may face us with no other alternative. Therefore, it is rational to use a few other compressors parallel. The number of these compressors might be determined based on the airflow needed and also the shortcoming in loss of air. In case of failure of one main compressor, one standby compressor will be replaced immediately to supply the same amount of compressed air, and it should be in use until the main one is repaired and then back to the system. Thus, determination of the reliability of the whole system is very important for product safety and economy and is highly dependent on the knowledge of the failure probability of compressors. Therefore, having a sufficient number of active and standby compressors increases the system efficiency regarding all the optimization matters.

Nowadays, numerous studies have been conducted on the energy saving and optimization of compressed air networks. Friedenstein et al.^2^ simulated the compressed air system of a gold underground mine in South Africa to identify the energy and operational improvement modifications in the compressed air systems. In this study, the refuge bay leaks were identified as a significant air user, and therefore, different scenarios were modeled by reducing the airflow to the refuge bay components. Results of this study showed that considering the optimized scenario improves the overall air usage and reduces the cost of electrical energy of mine, significantly^2^. Some similar research was done previously^3–5^. Fouché^6^ applied the control actions to improve the efficiency of the compressed air system in deep-level mining. In the reviewed study, control valves are used to fix leaks, adjust the delivery pressure of set points and accordingly reduce the air pressure on some levels. Results of this study showed that by the implementation of such actions, the electrical power consumption was reduced by 1.35 MW which had a considerable effect on the annual electricity cost saving. Chen et al.^7^ applied the general constrained nonlinear multi-objective model together with the critical path method to optimize the air quantity regulation in the mine ventilation networks. In this study, the main constraints were the upper and lower bounds of the air quantities of the branches and the pressure drops of the regulators. The proposed model was applied for two ventilation networks with single-fan and multi-fans. Results of the mentioned study stated that the proposed algorithm is so flexible and fast convergence that can be used for large-scale generalized ventilation networks. Hassan et al.^8^ tried to improve the compressed air usage in underground mines to reduce the electrical power consumption. In the reviewed study, different control techniques were proposed to reduce electrical energy consumption. These techniques were controlling the pressure of different set points to determine the minimum required air pressure for the mine shafts. The proposed techniques were applied in two deep gold mines in South Africa regarding their production conditions, infrastructure, and specifications. In another research Jacobs et al.^9^ tried to predict the failure of the centrifugal compressors. In the mentioned study, the Weibull distribution function was applied by using the leave-one-out cross-validation method to study the failure behavior of air compressors in a deep mine in South Africa. Zhang et al.^10^ proposed a fault diagnosis system for compressors. In this study, an algorithm based on the least squares supports vector machine was optimized using particle swarm optimization and then applied to create a fault diagnosis model. In similar research, the fault diagnosis for compressors was studied based on the back-propagation artificial neural network^11^, and hybrid deep belief network^12^, as well.

Reviewing the above-mentioned papers shows that most of the past studies have focused on reducing compressed air leakage, unauthorized compressed air usage, and also optimizing the airflow supply. These studies aimed to reduce the power consumption through optimization of the produced compressed air flow. But there is little reported work to analyze the availability and reliability of the compressed air supplying systems in the underground mining operation. During underground mining, the compressed air supplying system is required to be in operation continuously regarding the safety and technical issues. To reach this, a few numbers of standby compressors are required to be available to work as long as the unserviceable one is under repair process. Therefore, this paper is devoted to simulating all possible statuses for the compressed air supplying system to analyze the availability of each status. Regarding all these matters, deterministic approaches are worthless and the rational approach is a probabilistic one. Here is when the stochastic solution might come to help. Markov chain is a well-known powerful mathematical tool that is widely applied to generate a stochastic model of systems with sequence of possible states. This approach is based on the mathematical modelling in which the failure states are dependent only on the current state at that instance.

Markov modeling approach has great flexibility in expressing the dynamic behavior of systems. It can model most kinds of system behavior that can be modeled by combinatorial models suck as reliability block diagram and fault tree analysis. This approach can model different types of the systems’ behavior involving the complex repair, standby spares, sequence dependencies, and imperfect fault coverage that cannot be modeled by using the combinatorial models. Moreover, Markov modeling approach can be easily applied if the detailed representation of fault or error handling is required in the model^13^.

Nowadays, Markov chains modeling has been widely used for the system reliability and availability assessment in different fields of mining operations such as the reliability estimation of the auxiliary ventilation systems in the construction of long tunnels^14^, the selection of the crusher location^15^, production performance optimization of Load–Haul–Dump machines^16^, reliability analysis of mining drilling operation^17,18^, reliability, maintainability, and availability of tunnel boring machines^19,20^, and reliability, availability, and maintainability analysis of shovel and dump trucks in open pit mines^21^.

Ye et al.^22^ applied the continuous-time Markov chain for modeling the stochastic process of the failures and repairs of an air separation unit. In the mentioned study, two strategies were considered to increase the system availability. In the first strategy, parallel units were installed and in the second strategy, periodic inspections and maintenance were carried out. Then, a mixed-integer linear programming was proposed to optimize the selection of redundancy and the frequency of maintenance tasks for maximum profit. Rathi et al.^23^ studied the reliability of multistage reciprocating compressor systems using the Markov approach. In the reviewed paper, the state space model of all possible states (operating, standby, and failed) was created and then, the system reliability is estimated for different scenarios; with and without redundant compressors. Results of the mentioned study showed that the standby redundancy increased the reliability of the system. Liu et al.^24^ applied the Markov chain modeling to study the reliability of the mine ventilation systems. In the mentioned paper, the operation state of the system under the specified total operation time was simulated based on the Monte Carlo method, and then, the probability of a steady state in the future was analyzed. Zeqiri et al.^25^ studied the effectiveness of regulating ventilation systems in underground mines to ensure the circulation of the required amount of air through the workshops and mine facilities. In the mentioned study, different regulators and adjustments are used to reduce the amount of air in certain ways or even in different parts of the mine regarding the problem of ventilation of the underground mines. The results of the reviewed paper found the various methods of reliable regulation to ensure the designed amount of air with providing complete safety and comfort of microclimate during mining activity.

In this paper, a rigorous technique has been adopted from the Markov chain point of view to estimate the reliability of the compressed air supplying system when the number of both main and standby compressors and the other considerations like the probabilities of their failures and repair is distinct. The reliability of the system in different time steps is estimated and discussed, as well.

The paper is organized as follows. The research methodology and theoretical foundation of the paper are described in “Theoretical foundation” section. The case study and the state space diagram of the studied system are presented in “The case study; modeling of the compressed air supplying system” section. The results of the study are presented and discussed in “Reliability analysis of the compressed air supplying system” section.

## Theoretical foundation

This paper aims to analyze the reliability of the compressed air unit of Qaleh-Zari Copper Mine, Iran using the Markov modeling approach. In this section, the reliability analysis process through the Markov chain approach is presented. To achieve this, possible states for all main and reserve compressors are considered for the Markov chain modeling. The failure and repair rate of all compressors is calculated to obtain the probability of being of the system in each of the states. Then, the stationary state of the Markov chain is calculated to obtain the confidence interval for the system availability. Moreover, the probability of failure at any time period is considered to study the reliability behavior.

### Markov chain modelling and estimation of the transition probabilities

Markov chain is a stochastic process used for the mathematical modeling of a certain kind of phenomena depending on random variable. In this approach, the probability concepts are used to described how a system change from one state to the other states^26^. In this technique, the system does not have the memory. On the other hand, future states of a system depend on the current and the last immediate states. Furthermore, the behavior of the system must be the same at all points of time regardless of the point of time being considered. In this circumstance, the probability of transition from one state to another is constant at all times. On the other hand, the process is stationary or homogeneous^27^.

In the application of Markov modeling, system can be in only one state at a time, and from time to time it makes a transition from one state to another state. Two types of models can be considered for the modeling that are discrete- and continuous- time chains. In the discrete time Markov chains, transitions occur only at a fixed unit time interval with a transition required at each interval while, in the continuous time chains, the transitions are permitted to occur at any real-valued time interval. It is noted that the discrete case generally is known as a Markov chain and the Markov process is generally known for the continuous one^13^.

**Figure 1:**
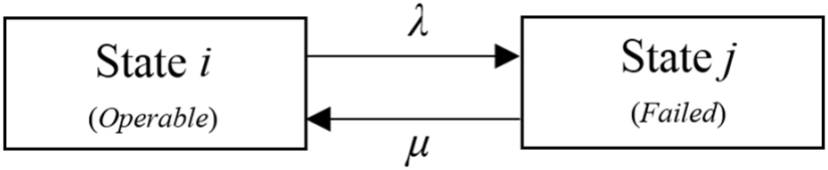
Basic Markov model.

At the first step of the Markovian approach, all states of the system are determined and the transfer rates (failure or repair) between states are obtained. Then, the Markov transition diagram is used to describe the relationship between the states of the system. Figure 1 shows a basic model of Markov chain with two states *i* and *j*, where *λ* and *μ* are the constant failure and repair (transfer rates), respectively. It notes that a chain in which every state can be reached from all other states either directly or indirectly through intermediate states is known as ergodic. In the ergodic Markov chains, the limiting values of the state probabilities are independent of the initial conditions^27^.

With considering the transition probability from each state *i* to state *j*, the probability that the system will be in state *j*, that it started in state *i* after *n* time intervals, can be calculated and discussed. The stochastic process {*X*_*n*_}, *n* = 0, 1, 2, …, is a discrete-time Markov chain for all states *i*_0_, … ,*i*,*j* if it satisfies the Markov property as^28–30^:

1 $$P(X_{n + 1} = j{|}X_{n} = i, X_{n - 1} = i_{n - 1} , \ldots , X_{0} = i_{0} ) = P(X_{n + 1} = j{|}X_{n} = i) = P_{ij}$$

where *P*_*ij*_ is the probability that the chain, whenever in state *i*, moves next (one unit of time later) into state *j*, $i \ne j$, and is referred to as a one-step transition probability. The $P_{ij}^{\left( n \right)}$ called the *n*-step transition probabilities as follows:

2 $$P_{ij}^{\left( n \right)} = P(X_{n} = j{|}X_{0} = i)$$

The $P_{ij}^{(n)}$ is the *n*-step transition probability. This means the probability that a process in state *i* will be in state *j* after *n* additional transitions. If the *n*-step transition probabilities are collected into the matrix form as $P(n) = \left\{ {p_{ij}^{(n)} } \right\}$, then according to Chapman-Kolmogorov equation, then $P^{\left( n \right)}$ equals $P^{n}$ for the stationary Markov chains.

The following stochastic constraints to the matrix are considered for the matrix *P*:

3 $$p_{ij} \ge 0,\quad \mathop \sum \limits_{j = 0}^{J} p_{ij} = 1,\quad i,j = 1, \ldots ,J$$

It worth to note that, regarding principle of the Markov chain process, the transition events are independent of one another and then, the transition probabilities ($p_{ij}$) can be obtained from the Binomial distribution.

### Estimating overall transition matrix

As mentioned, $P_{ij}^{n}$ is considered as the probability that the chain goes from state *i* to the *j* in *n*-steps and the new $P_{ij}^{n}$ numbers are arranged the entries of a matrix, named the *n*-step transition probability matrix or the overall transition matrix ($P^{n}$). The matrix $P^{n}$ is estimated through matrix multiplication. On the other hand, the *n*-step transition matrix can be obtained by multiplying the matrix *P* by itself *n* times. To do this, the eigenvalue and eigenvector approach, can be used. In this approach, the matrix *P *can be expanded as^30^;

4 $$P = U\Lambda U^{ - 1}$$

where Λ is the diagonal matrix of eigenvalues and *U* is the matrix whose columns are the corresponding eigenvectors. The, the overall transition matrix $P^{n}$ can be estimated from the following equation^29,30^:

5 $$P^{n} = U\Lambda^{n} U^{ - 1}$$

The remain of this subsection is devoted to study the long-term behavior of Markov chains. In should be noted that if the initial probability distribution of the Markov chain is known, then the probability distribution at some time instant *n* or $P_{ij}^{n}$ can be evaluated. For an ergodic Markov chain, the matrix multiplication technique can be applied to obtain the steady-state or the limiting values probabilities. The sequence of *n*-steps transition matrices $P^{n}$ approaches to a matrix whose rows are all identical. This means that, the $Lim_{n \to \infty } P_{ij}^{n} = \pi_{j}$. This indicates that, $P_{ij}^{n}$ is converging to some value which is the same for all *i*. On the other hand, a limiting probability exists that the process will be in state *j* after a large number of transitions, and this value is independent of the initial state *i*^29,30^. Either $\pi_{j}$ is the steady state distribution of the Markov chain. The steady state of a Markov chain can be obtained from the following equations:

6 $$\pi_{j} = \mathop \sum \limits_{i = 1}^{\infty } \pi_{i} P_{ij } ,\quad \mathop \sum \limits_{j = 1}^{\infty } \pi_{j} = 1,\quad j \ge 1$$

Consider *D* = {*S*_*d*_} as a set of desirable states and *U* = {*S*_*u*_} as a set of undesirable states. The mean time of being of the system in a set of desirable states ($\overline{D}$) desirable states ($\overline{U}$) and can be obtained from Eqs. (7) and (8), respectively^31^:

7 $$\overline{D} = \frac{{\mathop \sum \nolimits_{j \in D} \pi_{i} }}{{\mathop \sum \nolimits_{j \in U} \left( {\mathop \sum \nolimits_{j \in D} \pi_{i} .p_{ij} } \right)}}$$

8 $$\overline{U} = \frac{{\mathop \sum \nolimits_{j \in U} \pi_{j} }}{{\mathop \sum \nolimits_{j \in U} \left( {\mathop \sum \nolimits_{j \in D} \pi_{i} .p_{ij} } \right)}}$$

Moreover, the transition probability from desirable states to undesirable ones ($\overline{P}$) is obtained as:

9 $$\overline{P} = \frac{1}{{\overline{D} + \overline{U}}}$$

### Reliability and remaining lifetime estimation

The probability that the system will be in state *j*, that it started in state *i* for the first time (*n* = 1) between *m*-1 and *m* time steps, is obtained from the transition probability matrix as follows^30,31^:

10 $$f_{ij}^{\left( m \right)} = P(X_{n + m} = j, X_{n + m - 1} = i, \ldots , X_{n + 1} = i{|}X_{n} = i) = P_{ii}^{m - 1} P_{ij}$$

The same approach can be considered for different time step and the cumulative probability function can be obtained. Therefore, the reliability function of the event at time *τ*, where the system remains in state *j* which it started in state *i* for the first time (*n* = 1) is formulated from the following equation:

11 $$R(\tau ) = 1 - F_{ij} (\tau ) = 1 - \mathop \sum \limits_{m = 1}^{\tau } P_{ii}^{m - 1} P_{ij} ,\quad \tau = 1,2, \ldots$$

where $F_{ij} (\tau )$ in the cumulative transition probability.

In this approach, the lifetime probability of transitioning from state *i* to state *j* can be calculated, as well. The expected value for the first transition from *i* to *j* is formed as:

12 $$\mathop \sum \limits_{r = 1}^{\infty } mf_{ij}^{(m)} = 1$$

Then, the expected time to the state *j* is calculated as:

13 $$E_{ij} (\tau ) = \frac{{\mathop \sum \nolimits_{r = 1}^{\infty } mf_{ij}^{(m)} }}{{1 - p_{ii}^{m} }}|_{r = 1} = \frac{1}{{1 - p_{ii} }}$$

## The case study; modeling of the compressed air supplying system

Qaleh-Zari Copper Mine from Minakan Company is located 180 km from Birjand city in Southern Khorasan Province, Iran. The mine is located at the coordinates of 57′ 58° geographical longitude and 31′ 48° geographical latitude. Qaleh-Zari copper mine is the only underground mine in Iran that is extracted by shrinkage-stopping methods. The grade of copper, gold, and silver is 0.5–8%, 0.5–15, and 20–600 g per tonne, respectively^32,33^. Moreover, the total extracted and remaining deposits equal about 10 million tons. The width of the mineralization area is between 0.5 and 7 m which is extracted by using the drilling and blasting method. The blast holes are drilled by using the pneumatic drilling pickers. The pneumatic loaders are used to load the extracted ores into wagons. The extracted ores are then moved to the surface through six vertical shafts and one inclined (main) shaft with a total production of 450 tonnes per day, on average. All shafts have their own compressor house to handle all pneumatic equipment. It should be noted that the compressor house of the main shaft (the incline shaft) not only handles the pneumatic equipment in the underground mining operation but also support the mineral prospecting plant.

**Figure 2:**
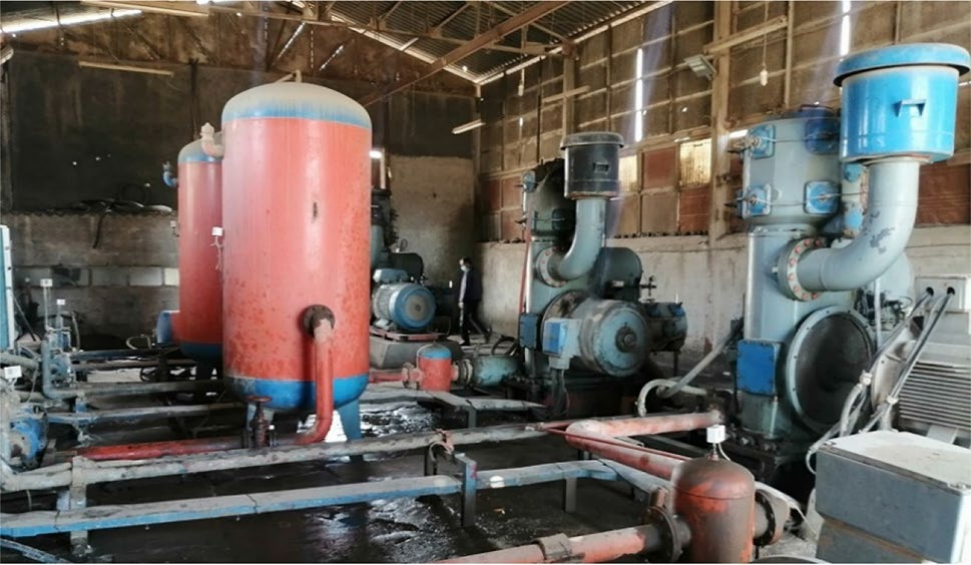
The main compressor house of the Qaleh-Zari Copper Mine.

This paper aims to apply an approach to evaluate the reliability of the compressor house of the inclined shaft of the mine using the stochastic approach. Therefore, having the failure and repair rate of the compressors and the knowledge about the event probabilities are necessary. The reliability of the compressed air unit is estimated using the Markov chains theory in which the probability of failure or a repair is not dependent on the past history of the system. The compressed air system of the main shaft of the Qaleh-Zari copper mine consists of two compressors as main compressors and one standby compressor that works in three working shifts and 30 days per month or 720 h (= 30 × 24) each month. If any main compressor failed, due to the failure of different or any other causes, the reserve compressor is replaced immediately and the failed compressor will be repaired. The main compressor house of the Qaleh-Zari Copper mine is shown in Fig. 2.

The production capacity of all main compressors is about 30 cubic meters per hour. Regarding the statistical analysis, each main compressor is failed about 2 times in month and repair of any failed compressor is taken 17.5 h in average. So, the failure probability of main compressors in each month (or in 720 h per month (= 30 day/mouth × 24 h/day)) is estimated as ((2 × 17.5)/720 =) 0.049. Similarly, the probability of failure of standby compressors is calculated as 0.038. The main failure of the compressors was related to the electromotor, air filter, and drain receiver tank. The probability of repairing each compressor is estimated as 0.973, as well. Moreover, the statistical analysis showed that the electric current is off for 6 h per month, on average, and then, the probability of electric blackout is calculated as 0.008 (= 6/720).

Considering the proposed concept in “Theoretical foundation” section, by calculating the probability of occurrence possessions in which all of the main compressors were replaced with standby compressors and all of the main compressors have failed, the reliability of the compressed air supplying system can be estimated.

**Figure 3:**
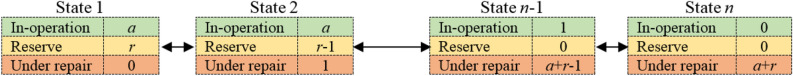
Operation condition of the compressors.

Suppose that a compressed air system has ‘‘*a*’’ active and ‘‘*r*’’ reserve or standby compressors where *a* ≥ *r*. When an active compressor is failed, one of the reserve compressors is replaced with the failed one and it continues to operate as long as the failed compressor is repaired and back to the compressed air circuit. The process will be forward until all “*r*” reserve compressors are replaced by “*r*” out of “*a*” in-operation compressors and will be run in the backward direction when a failed compressor is repaired. This process will be continued until all main and reserve compressors are failed. In this situation, the number of failed compressors increased to “*a* + *r*” and there is no in-operation compressor. This process is shown as a state space diagram for “*a*” in-operation and “*r*” reserve compressors in Fig. 3. Regarding Fig. 3, the movement between states occurs in discrete time steps and the system is homogeneous. Therefore, this can be considered a discrete Markov process.

The remaining of this section is devoted to the use of the Markov chain process for modeling the compressed air system. In this section, the stochastic process, described in “Theoretical foundation” section, is used to model and analyze the compressed air system in the Qaleh-Zari copper mine.

**Figure 4:**
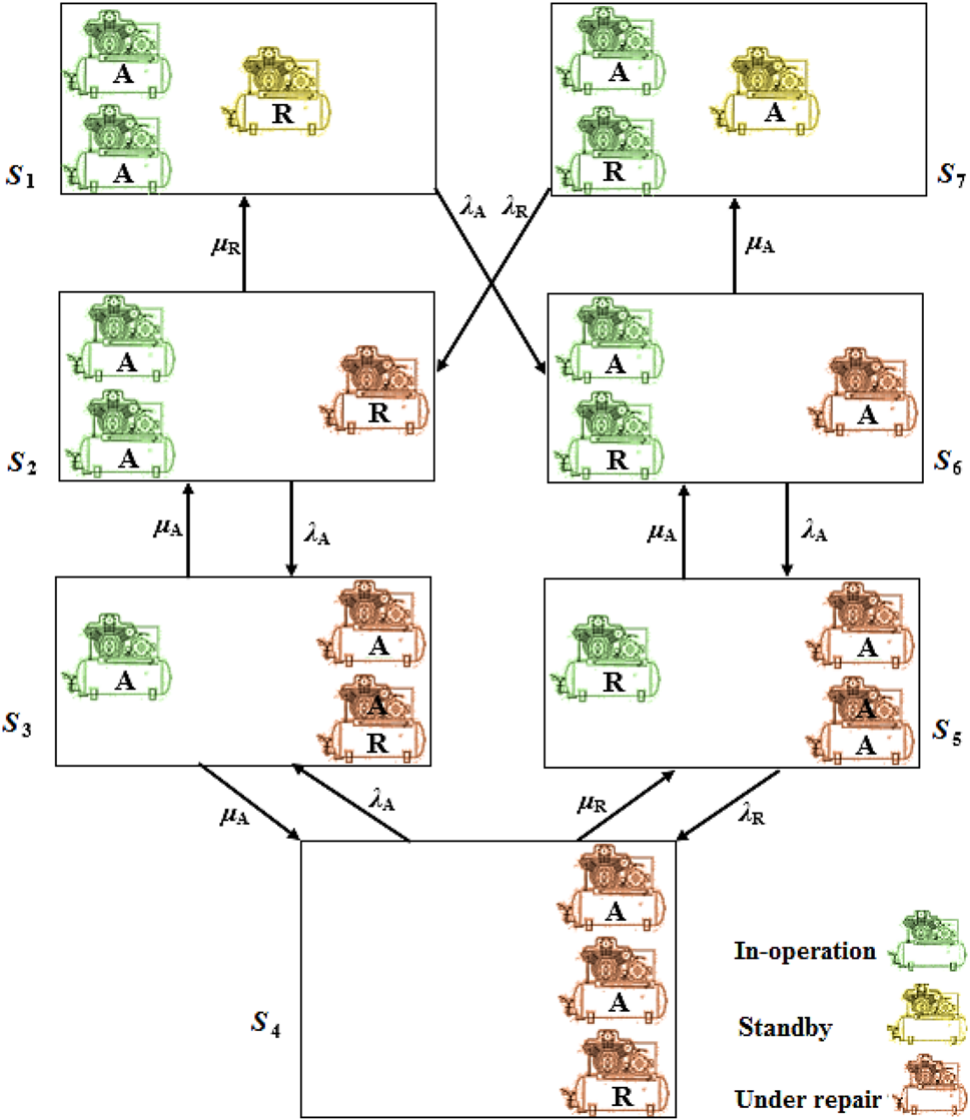
All possible states for the in-operation, standby and under-repair compressors.

Different stages for all three compressors in the main compressed air system of the mine are considered and shown in Fig. 4. In Fig. 4, in the normal operating condition, there are two main compressors (shown by “A”) and one reserve compressor (shown by “R”) in the mine compressor house. Regarding Fig. 4, in *S*_1_ and *S*_7_ states, all of the two compressors are in operation and there is one standby compressor. *S*_2_ and* S*_6_ are the states in which one of the two active or the standby compressors are out of work. Therefore, in this position, one of the compressors (main or reserve) is in under-repair operation. In states *S*_3_ and *S*_5_, two compressors are out of work and only one compressor is active. Finally, in the *S*_4_ state, all compressors failed and no one is active or on standby condition. The above-mentioned states, *S*_1_ to *S*_7_, construct a Markov chain and therefore the probability of the condition change of each compressor from one state to the alternative one can be calculated.

To illustrate the process, the following example shows what happens when the system goes from state one (*S*_1_) to state six (*S*_6_). It means that one of the main compressors failed and then it is repealed by the reserve compressor. The probability of this event is calculated by Bernoulli Distribution as described here. As mentioned earlier, the failure probability of the main compressors and the activation probability of the reserve compressor are 0.057 and 0.943 (= 1–0.057), respectively. Therefore, the probability of going from state one to state six is calculated:

14 $$P_{1 \to 6} = \left( {\begin{array}{*{20}c} 2 \\ 1 \\ \end{array} } \right) \times 0.057 \times \left( {1 - 0.057} \right)^{2 - 1} \times 0.943 = 0.101$$

It notes that *P*_1→6_ is considered for the sixth array of the first row of the transition matrix. Moreover, all rows of this matrix should obey the Markov chain analysis. This means that the summation of the probabilities in each row equals 1. Hence, there is no transition from state one to another, then *P*_1→1_ is calculated as 0.899 (= 1 − 0.101). Similarly, the probability of transmission system from one state to another has been calculated and arrayed to the transition matrix. Regarding the above-described calculations, the transition matrix (*P*) for the compressed air supplying system is constructed and given as Eq. (15).

15 $$P = \left[ {\begin{array}{*{20}c} {0.899} & 0 & 0 & 0 & 0 & {0.101} & 0 \\ {0.973} & {0.024} & {0.003} & 0 & 0 & 0 & 0 \\ 0 & {0.026} & {0.974} & 0 & 0 & 0 & 0 \\ 0 & 0 & {0.001} & {0.973} & {0.026} & 0 & 0 \\ 0 & 0 & 0 & {0.001} & {0.946} & {0.053} & 0 \\ 0 & 0 & 0 & 0 & {0.047} & {0.063} & {0.891} \\ 0 & {0.046} & 0 & 0 & 0 & 0 & {0.954} \\ \end{array} } \right]$$

## Reliability analysis of the compressed air supplying system

This section is devoted to estimating the reliability and accordingly the expected time to failure for the compressed air supplying system. To achieve this, first, the *P*^*n*^ transition probability matrix was estimated from Eq. (6). The overall transition matrix is given in Eq. (16). It is clear that *n* = 1 from the *P*^*n*^ matrix gives the actual first transition matrix.

16 $$P^{n} = \left[ {\begin{array}{*{20}c} {0.033( - 0.03^{n} ) - 0.055(0.12^{n} ) + 0.741(0.84^{n} )} & \cdots & {0.034( - 0.03^{n} ) + 0.094(0.12^{n} ) - 0.751(0.84^{n} )} \\ { - \,0.004(0.95^{n} ) + 0.001(0.97^{n} ) + 0.284} & {} & { - \,0.004(0.95^{n} ) + 0.004(0.97^{n} ) + 0.622} \\ \vdots & \ddots & \vdots \\ {0.029( - 0.03^{n} ) + 0.030(0.12^{n} ) - 0.367(0.84^{n} )} & \cdots & {0.031( - 0.03^{n} ) - 0.051(0.12^{n} ) + 0.372(0.84^{n} )} \\ { + \,0.022(0.95^{n} ) + 0.002(0.97^{n} ) + 0.284} & {} & { + \,0.022(0.95^{n} ) + 0.005(0.97^{n} ) + 0.622} \\ \end{array} } \right]$$

The sequence of *n*-steps transition matrixes,* P*^*n*^, approaches the stationary matrix, in which its rows are the unique fixed probability vector; hence, the probability $P_{ij}^{n}$ that *S*_*j*_ occurs for sufficiently large *n* is independent of the original state *S*_*i*_ and it approaches the component *f*_*j*_ of *F.* The stationary matrix is formed by *P* power to a large number, indicating a very quick convergence. The stationary matrix is given in Eq. (17).

17 $$F = \left[ {\begin{array}{*{20}c} {0.284} & {0.029} & {0.003} & {0.001} & {0.028} & {0.032} & {0.622} \\ {0.284} & {0.029} & {0.003} & {0.001} & {0.028} & {0.032} & {0.622} \\ {0.284} & {0.029} & {0.003} & {0.001} & {0.028} & {0.032} & {0.622} \\ {0.284} & {0.029} & {0.003} & {0.001} & {0.028} & {0.032} & {0.622} \\ {0.284} & {0.029} & {0.003} & {0.001} & {0.028} & {0.032} & {0.622} \\ {0.284} & {0.029} & {0.003} & {0.001} & {0.028} & {0.032} & {0.622} \\ {0.284} & {0.029} & {0.003} & {0.001} & {0.028} & {0.032} & {0.622} \\ \end{array} } \right]$$

On the other hand, since the process of the compressed air supplying system is modeled by an ergodic Markov chain, the stationary state of the Markov chain can be obtained by using Eq. (6), as well:

18 $$(\pi_{1} ,\pi_{2} , \ldots , \pi_{7} ) \times P = (\pi_{1} ,\pi_{2} , \ldots , \pi_{7} ), \mathop \sum \limits_{i = 1}^{7} \pi_{i} = 1$$

where $\pi_{i}$ is the probability of the event that the system remains in the state *S*_*i*_. Regarding Eq. (16), the $\pi_{i}$ values are obtained as follows:

$$\pi_{{1}}$$ = 0.284, $$\pi_{2}$$ = 0.029, $$\pi_{3}$$ = 0.003, $$\pi_{4}$$ = 0.001, $$\pi_{5}$$ = 0.028,$$\pi_{6}$$ = 0.032, $$\pi_{7}$$ = 0.622.

These results indicate that there is 28.4% probability that the compressed air supplying system in the Qaleh-Zari mine is in operating condition with two main and one standby compressors at any proposed time. On the other hand, the confidence interval for availability of two main compressors and one standby compressor equals to 28.4%.

Considering *S*_1_ and *S*_7_ as the desirable states in which there are two active compressor and one standby compressor and states *S*_2_ and *S*_6_ as undesirable states, the mean time of being of the system in these statuses is obtained from Eqs. (7) to (9) as follows:

19 $$\overline{D} = \frac{0.284 + 0.621}{{(0.284 \times 0.101) + (0.621 \times 0.054)}} = 14.55$$

20 $$\overline{U} = \frac{0.029 + 0.003 + 0.001 + 0.028 + 0.032}{{(0.029 \times 0.973) + (0.003 \times 0) + (0.001 \times 0) + (0.028 \times 0) + (0.032 \times 0.875)}} = 1.66$$

21 $$\overline{P} = \frac{1}{14.55 + 1.66} = 0.062$$

**Figure 5:**
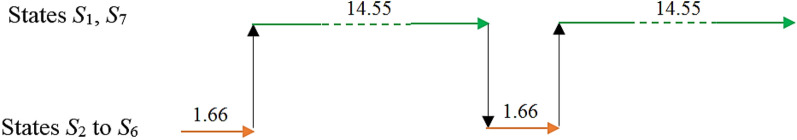
The mean time of being of the system in desirable and undesirable states.

Regarding the results, compressed air supplying system is in the desirable states (*S*_1_ and *S*_7_) or have two active and one standby compressors in 14.55 of the time steps, on average. In only 1.66 of the time steps, the system is in the undesirable states. These results can be graphically shown in Fig. 5.

The transition probability from desirable states to undesirable ones is calculated as 0.06. By considering the number of 30 days as the total working days in each month, it is concluded that the two active and one standby compressor are available for ((1 − 0.062) × 30≈) 28 days a month. These results indicate that the compressed air supply system in Qaleh-Zari copper mine is in a high availability level.

Remaining of this section is devoted to estimate the system reliability. To achieve this, first the probability that a first failed compressor is replaced by a reserve compressor is estimated using Eq. (10). The first transition probabilities across various time steps from 1 to 20 are obtained and given in Table 1. Regarding Table 1, the probability that the compressed air supplying system has two main compressors without failure for one month is 91.29% (= 1 − 0.0871). It also takes about 7 months for the compressed air supplying system that the first failure probability reaches 50%.

**Table 1:** Probability of the first failure in different time steps.

Time step	Failure probability	Time step	Failure probability
1	0.1010	11	0.0422
2	0.0908	12	0.0401
3	0.0816	13	0.0383
4	0.0734	14	0.0368
5	0.0664	15	0.0355
6	0.0605	16	0.0344
7	0.0555	17	0.0335
8	0.0513	18	0.0328
9	0.0478	19	0.0321
10	0.0448	20	0.0316

**Figure 6:**
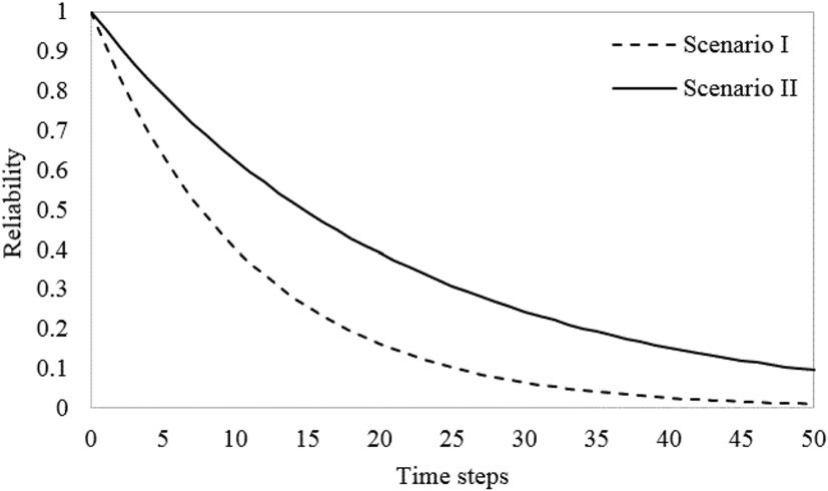
System reliability with two active and one standby compressors in the form of two Scenarios.

According to Fig. 4, in the states *S*_1_ and *S*_7_ two compressors are in operation and there is one standby compressor. By using the same procedure, mentioned earlier, the failure probability and consequently the reliability of the system’s statuses in which there are two in-operation compressors and one standby compressor at any time step were calculated in two scenarios. In scenario I (tarnation from *S*_1_ to *S*_6_) two main compressors are in operation and in scenario II (tarnation from *S*_7_ to *S*_2_) one main and one reserve compressors are active. The results are shown in Fig. 6. Regarding Fig. 6, the system probability that two compressors are remain in the operation and one compressor being standby without failure for one months is 92.32% and 95.4% for scenario I and II, respectively. It can be stated that, after 50th time step (or 4 years after the study period), the probability of the first failure for the compressed air system with two main in-operation compressors and one standby compressor reaches one. This situation will be occurred after 110th time step (or approximately 9 years) for scenario II.

The expected time to the failure was also estimated from Eq. (13) for each status. The remaining life of the compressed air supplying system or the expected time to state 4 from states 3, and 5 were found to be 33 and 20 months, respectively. These estimates revealed that the average lifetime of the compressed air supplying system of the Qaleh-Zari copper mine is approximately 65 percent higher when at least one main compressor is active in comparison with the reserve one. This is because the main compressors are more reliable than the reserve ones.

## Conclusions

Most of the past studies have focused on reduction of the power consumption through optimization of the produced compressed air flow. However, there is little reported work to analyze the availability and reliability of compressed air systems in underground mining operations. In this paper, the availability and reliability of the compressed air system was analyzed through Markov Chain-Based Stochastic Modeling as a case study; Qaleh-Zari copper mine, Iran. To achieve this, first, the compressed air system was simulated by considering all possible states for the in-operation, standby, and under-repair compressors. Then, the sequences of *n*-steps transition matrixes were estimated and analyzed. Regarding the results, there is 91.29% probability for the compressed air supplying system of the mine to have at least two main compressors in the operation status for one month. Considering 30 days as the total working days of mine in each month, two in-operation compressors and one standby compressor will be available in 28 days. Moreover, the remaining life of the compressed air supplying system with two main compressors was found to be 33 months.

The results of this study open a new horizon for mine managers and contractors to have an available air supplying system to ensure the production capacity and select the most appropriate production scheduling program. Considering the appropriate inspection and maintenance intervals to have an efficient compressed air system and studying the effect of the environmental operating condition on the reliability and remaining life of the system are proposed for future studies.

## Data Availability

All data generated or analyzed during this study are included in this published article.

## References

[1] Schroeder, F. W. Energy *Efficiency Opportunities in Mine Compressed Air Systems*. Doctoral Dissertation, North-West University (2009).

[2] Friedenstein BM, Cilliers C, Van Rensburg J (2018). Simulating operational improvements on mine compressed air systems. S. Afr. J. Ind. Eng..

[3] Marais, J. & Kleingeld, M. Simplification of mine compressed air systems. In *Proceedings of the Industrial and Commercial Use of Energy Conference (ICUE), Cape Town, South Africa* (2013).

[4] Kriel, C. *Modernising Underground Compressed Air DSM Projects to Reduce Operating Costs*. MEng Dissertation, Dept. Mech. Eng., North-West University, Potchefstroom (2014).

[5] De Coning, A. *Sustained Energy Performance on Compressed Air Systems for Expanding Gold Mines*. Doctoral Dissertation (2013).

[6] Fouché, S. J. *Improving Efficiency of a Mine Compressed Air System*. Doctoral Dissertation, North-West University (South Africa), Potchefstroom Campus (2017).

[7] Chen K, Si J, Zhou F, Zhang R, Shao H, Zhao H (2015). Optimization of air quantity regulation in mine ventilation networks using the improved differential evolution algorithm and critical path method. Int. J. Min. Sci. Technol..

[8] Hassan, A., Ouahada, K., Marwala, T. & Twala, B. Optimization of the compressed air-usage in South African mines. In *IEEE Africon'11* 1–6 (IEEE, 2011).

[9] Jacobs JA, Mathews MJ, Kleingeld M (2019). Failure prediction of mine compressors. J. Fail. Anal. Prev..

[10] Zhang K, Su J, Sun S, Liu Z, Wang J, Du M, Liu Z, Zhang Q (2021). Compressor fault diagnosis system based on PCA-PSO-LSSVM algorithm. Sci. Prog..

[11] Zhou Z, Wang J, Chen H, Wei W, Xu C (2020). An online compressor liquid floodback fault diagnosis method for variable refrigerant flow air conditioning system. Int. J. Refrig.

[12] Tran VT, AlThobiani F, Tinga T, Ball A, Niu G (2018). Single and combined fault diagnosis of reciprocating compressor valves using a hybrid deep belief network. Proc. Inst. Mech. Eng. C J. Mech. Eng. Sci..

[13] Boyd, M. A. & Lau, S. An introduction to Markov modeling: Concepts and uses. In *Annual Reliability and Maintainability Symposium, Anahiem, USA* (1998).

[14] Jalali SE, Forouhandeh SF (2011). Reliability estimation of auxiliary ventilation systems in long tunnels during construction. Saf. Sci..

[15] Yarmuch J, Epstein R, Cancino R, Peña JC (2017). Evaluating crusher system location in an open pit mine using Markov chains. Int. J. Min. Reclam. Environ..

[16] Gustafson A, Lipsett M, Schunnesson H, Galar D, Kumar U (2014). Development of a Markov model for production performance optimisation. Application for semi-automatic and manual LHD machines in underground mines. Int. J. Min. Reclam. Environ..

[17] Ugurlu OF, Kumral M (2020). Reliability-based performance analysis of mining drilling operations through Markov chain Monte Carlo and mean reverting process simulations. Simulation.

[18] Rahimdel MJ, Hoseinie SH, Ghodrati B (2016). RAM analysis of rotary drilling machines. Min. Sci..

[19] Ahmadi S, Hajihassani M, Moosazadeh S, Moomivand H (2020). An overview of the reliability analysis methods of tunneling equipment. Open Constr. Build. Technol. J..

[20] Agrawal AK, Murthy VMSR, Chattopadhyaya S (2019). Investigations into reliability, maintainability and availability of tunnel boring machine operating in mixed ground condition using Markov chains. Eng. Fail. Anal..

[21] Kumar NH, Manjunath C, John RP, Chand RP, Madhusudhana S, Venkatesha BK (2022). Reliability, availability and maintainability study of 6.5 cubic meters shovel and 60 tone dumper in a surface limestone mine. Mater. Today Proc..

[22] Ye Y, Grossmann IE, Pinto JM, Ramaswamy S (2019). Modeling for reliability optimization of system design and maintenance based on Markov chain theory. Comput. Chem. Eng..

[23] Rathi P, Kumar G, Asjad M, Soni U (2022). Reliability improvement of a multistage reciprocating compressor with redundancies using Markov approach. J. Ind. Integr. Manag..

[24] Liu L, Liu J, Zhou Q (2022). Mine ventilation system reliability evaluation based on a Markov chain. Sci. Rep..

[25] Zeqiri I, Gashi J, Brahimaj F, Zeqiri R (2022). Effectiveness of ventilation regulation in a simple diagonal system of underground mines. Min. Miner. Depos..

[26] Chan, K. C., Lenard, C. T. & Mills, T. M. An introduction to Markov chains. In *49th Annual Conference of Mathematical Association of Victoria* 40–47 (2012).

[27] Billinton R, Allan RN (1992). Reliability Evaluation of Engineering Systems.

[28] Mantyla, V. M. Discrete hidden Markov models with application to isolated user-dependent hand gesture recognition, Vol. 4, no. 4 9 (VTT Publications, 2001).

[29] Ross SM (2014). Introduction to Probability Models.

[30] Miller S, Childers D (2012). Probability and Random Processes: With Applications to Signal Processing and Communications.

[31] Serfozo R (2009). Basics of Applied Stochastic Processes.

[32] Afradi A, Alavi I, Moslemi M (2021). Selecting the best mining method using analytical and numerical methods. J. Sediment. Environ..

[33] Rahimi Ghazikalayeh A, Ebrahimabadi A, Alavi I (2014). Selecting proper mining method using fuzzy AHP approach (case study: Qaleh-Zari Copper Mine of Iran). J. Appl. Sci. Agric..

